# Fucoxanthin, the constituent of *Laminaria japonica*, triggers AMPK-mediated cytoprotection and autophagy in hepatocytes under oxidative stress

**DOI:** 10.1186/s12906-018-2164-2

**Published:** 2018-03-20

**Authors:** Eun Jeong Jang, Sang Chan Kim, Ju-Hee Lee, Jong Rok Lee, Il Kon Kim, Su Youn Baek, Young Woo Kim

**Affiliations:** 1 College of Oriental Medicine, Daegu Haany University, Gyeongsan, Gyeongsangbuk-do 38610 South Korea; 2 College of Korean Medicine, Dongguk University, Gyungju, Gyeongbuk 38066 South Korea; 3 Kyungpook National University, Daegu, 41566 South Korea

**Keywords:** Fucoxanthin, Oxidative stress, AMPK, Autophagy, AMPK/mTOR/ULK-1 pathway

## Abstract

**Background:**

*Laminaria japonica* has frequently been used as a food supplement and drug in traditional oriental medicine. Among the major active constituents responsible for the bioactivities of *L. japonica,* fucoxanthin (FX) has been considered as a potential antioxidant. This study was conducted to examine the effects of *L. japonica* extract (LJE) or FX against oxidative stress on hepatocytes and to elucidate the overall their cellular mechanisms of the effects.

**Methods:**

We constructed an in vitro model with the treatment of arachidonic acid (AA) + iron in HepG2 cells to stimulate the oxidative damage. The cells were pre-treated with LJE or FX for 1 h, and incubated with AA + iron. The effect on oxidative damage and cellular mechanisms of LJE or FX were assessed by cytological examination and several biochemical assays under conditions with or without kinase inhibitiors.

**Results:**

LJE or FX pretreatment effectively blocked the pathological changes caused by AA + iron treatment, such as cell death, altered expression of apoptosis-related proteins such as procaspase-3 and poly (ADP-ribose) polymerase, and mitochondria dysfunction. Moreover, FX induced AMPK activation and AMPK inhibitor, compound C, partially reduced the protective effect of FX on mitochondria dysfunction. Consistent with AMPK activation, FX increased the protein levels of autophagic markers (LC3II and beclin-1) and the number of acridine orange stained cells, and decreased the phosphorylation of mTOR and simultaneously increased the phosphorylation of ULK1. And the inhibition of autophagy by 3-methylanine or bafilomycin A1 partially inhibited the protective effect of FX on mitochondria dysfunction.

**Conclusion:**

These findings suggest that FX have the function of being a hepatic protectant against oxidative damages through the AMPK pathway for the control of autophagy.

## Background

*Laminaria japonica*, one of the most well known brown seaweeds, is referred to as “Dashima” in Korean, “Kombu” in Japanese, and “Haidai” in Chinese. *L. japonica* is widely used as a food supplement, as well as a drug for treatment of various diseases [1]. *L. japonica* has abundant bioactive components, including polyphenols, pigments, polysaccharides, minerals and amino acids [1]. Among bioactive compounds in *L. japonica*, fucoxanthin (FX), a marine carotenoid, has remarkable biological properties, including anti-cancer, obesity and inflammation [1–4]. FX has attracted much attention as a potential antioxidant given its unique chemical structure (Fig. 2a), which includes an allenic bond, epoxide group, and hydroxyl group [5]. Liu et al. (2011) reported that FX significantly recovered cell proliferation and increased the levels of glutathione and decreased intracellular reactive oxygen species (ROS) induced by ferric nitrilotriacetate [6]. In recently, Seo et al. (2016) showed that FX inhibited lipid accumulation and ROS formation by controlling adipogenic and lipogenic factors and ROS-regulating enzymes during differentiation in 3 T3-L1 adipocytes [7]. It is indicated that FX can effectively protect against hepatotoxicity by reducing intracellular ROS, associated with the antioxidant effects of FX.

ROS is a group of molecules including superoxide anion, hydroxyl radical and hydrogen peroxide, mainly produced in the mitochondria [8]. However, excess ROS can be involved in oxidative stress that destroys the structure of vital biomolecules, potentially leading to cellular dysfunction and remodeling [9]. Oxidative stress is known to activate the AMP-activated protein kinase (AMPK) signaling system in neuronal, heart, muscle, pancreatic and liver cells [10, 11]. Interestingly, AMPK is known to be involved in ROS-induced autophagy that promotes cell survival in response to cellular stress such as malnutrition, hypoxia or ischemia [12]. Indeed, oxygen and nutrient deprivation induce the activation of AMPK leading to autophagy by inhibition of mTORC1 and phosphorylation of ULK1 [13, 14].

Autophagy is an important cell pathway for cell homeostasis and survival by removing damaged organelles and intracellular microbial pathogens [15]. Hepatocytes may be particularly dependent on the underlying autophagy for normal physiological function due to their high biosynthetic activity. In addition, autophagy plays a crucial role in the non-alcoholic and alcoholic liver diseases, drug-induced hepatic damage, viral hepatitis, fibrosis, liver cancer and hepatic ischemia reperfusion injury [15–17]. In liver ischemia reperfusion injuries, autophagy provides a prosurvival activity allowing the cell to cope with nutrient starvation and anoxia [16]. During hepatitis B or C infection, the level of autophagy is typically increased to promote viral growth [17]. In hepatocellular carcinoma, the level of autophagy is thought to be involved in both tumorigenesis and tumor suppression [18, 19].

In this regard, we tested whether *L. japonica* and FX alleviated hepatic oxidative stress in an in vitro model, HepG2 cells established by arachidonic acid (AA) + iron. Specifically, we explored the abilities of FX in regulation of autophagy and the underlying molecular mechanisms of their effects.

## Methods

### Reagents

AA and Compound C (C.C) were purchased from Calbiochem (San Diego, CA, USA). Anti-phospho-ACC, phospho-LKB1, procaspase-3, PARP, Bcl_XL,_ LC3 I/II, beclin-1, AMPK, and phospho-AMPK antibodies were obtained from Cell Signaling Technology (Beverly, MA, USA). Bal-A1 was purchased from Santa Cruz Biotechnology (Santa Cruz, CA, USA). Horseradish peroxidase-conjugated goat anti-rabbit, rabbit anti-goat, and goat anti-mouse IgGs were obtained from Zymed Laboratories (San Francisco, CA, USA). FX, acrydine orange hemi zinc chloride salt, 3-methyladenine (3-MA), anti-ß-actin antibody and other reagents were purchased from Sigma-Aldrich (St. Louis, MO, USA).

### Preparation of the *L. japonica* extract *(LJE)*

*L. japonica* was purchased from Daewon pharmacy (Daegu, Korea), which is standardized with a standard herb of *L. japonica* in Korea Food and Drug Administrations. The *L. japonica* (100 g) were extracted as previously described [20, 21]. The yield of lyophilized LJE was estimated to be 1.19% based on the dried weight.

### Cell culture

HepG2 cells, a human hepatocyte-derived cell line, were provided by American Type Culture Collection (Rockville, MD, USA), and cultured as previously described [20]. To simulate oxidative stress, cells were incubated with 10 μM AA for 12 h, followed by exposure to 5 μM iron for 1 h. The cells were treated with FX or LJE for 1 h prior to the incubation with AA at the indicated doses.

### Cell viability assay

The cells were plated at a density of 1 × 10^5^ cells per well in a 48-well for 24 h as previously described [20]. The media was incubated with 0.25 mg/ml MTT for 2 h, and formazan crystals were dissolved with the addition of 200 μl DMSO.

### Terminal deoxynucleotidyl transferase dUTP nick end labeling (TUNEL) assay

The TUNEL assay was performed using the DeadEnd™ Colorimetric TUNEL System, according to the manufacturer’s instruction. The samples were washed and examined under light microscope.

### Western blot analysis

The cells were plated at a density of 5 × 10^5^ cells per well in a 6-well plate for 24 h. After the treatment designated, cells were lysed in RIPA buffer (Thermo Scientific, Rockford, IL, USA) as previously described [20, 21]. The protein bands were detected using Fusion Solo scanning system (Vilber Lourmat, Paris, France), and quantified using Image J ver 1.42 software (NIH, Bethesda, USA).

### Measurement of ROS production

DCFH-DA, a cell-permeable non-fluorescent probe, has been used as a substrate for quantitation of intracellular oxidant production in HepG2 cells [21]. After treatment of reagents, cells were stained with 10 μM DCFH-DA for 30 min at 37 °C. The fluorescence intensity in the cells was measured at an excitation/emission wavelength of 485/535 nm, using in the cells measured in a microplate reader.

### Determinant of glutathione (GSH) content

Intracellular GSH content was quantified using a commercial GSH BIOXYTECH GSH-400 kit (Oxis International, Portland, OR) according to the manufacturer’s protocol, and the absorbance level was measured at 405 nm.

### Flow cytometric analysis of ΔΨm

ΔΨm was measured using rhodamine123 (Rh123). Following treatment, cells were stained with 0.05 μg/ml of Rh123 for 1 h and then harvested by trypsinization. The change in ΔΨm was monitored using a FACS flow cytometer (Partec, Münster, Germany). In each analysis, a total of 10,000 events were recorded as previous described [20].

### Acridine orange (AO) staining

HepG2 cells were plated on 18-mm cover glasses and incubated for 24 h to reach at approximately 70% confluence. They were then incubated either in the presence or absence of 30 μM FX, washed twice with PBS and fixed with ice-cold 4% paraformaldehyde for 10 min at room temperature. Subsequently, the cells were stained with AO (1 μg/ml) for 15 min at room temperature, washed, and examined under a fluorescence microscope (Nikon, Japan).

### Profiling the content of fucoxanthin by ultra performance liquid chromatography (UPLC)

Water ACQUITYTM ultraperformance LC system (USA) was used to assess UPLC analysis as previously described [20]. Waters ACQUITYTM BEH C18 column (1.7 μm, 2.1 mm × 100 mm) was used as Waters ACQUITYTM PDA and HPLC Column, and the fucoxanthin was analyzed at 330 nm. The standard fucoxanthin was melted by methanol and diluted to make solution containing 1 μg/ml. *L. japonica* 1 g was also added with methanol 10 ml, and then sonication was perfomed for 3 h. After then, it was filtered through a 0.2 μm filter (Nalgene, NY, USA). A mobile phase was a mixed liquid of the acetonitrile and water, and the analysis condition was as in Table 1. The sample was injected with 2 μl, and a flow rate was 0.4 ml/min.

**Table 1:** Solvent gradient for the analysis fucoxanthin in *L. japonica*

Time (min)	0.1% FA/ Water (%)	0.1% FA/ Acetonitrile (%)	Flow rate (ml/min)
0	98	2	0.40
1	98	2	0.40
2	90	10	0.40
4	70	30	0.40
7	50	50	0.40
9	30	70	0.40
10	10	90	0.40
12	0	100	0.40
14	98	2	0.40
16	98	2	0.40

### Statistical analysis for study

GraphPad Prism software version 5.01 (Graph Pad Software, La Jolla, CA) was used for all statistical analyses as previously described [20, 21]. Significance levels were calculated by repeated measures of ANOVA with the Dunnett post hoc test under 95% confidence interval. Data were presented as the mean with standard deviation (mean ± S.D.). Within figures, the *P* values were displayed with asterisks (***P < 0.001, **P < 0.01, *P < 0.05).

## Results

### *L. japonica* Extract (LJE) decreases AA + iron induced cytotoxicity in HepG2 cells

**Fig. 1:**
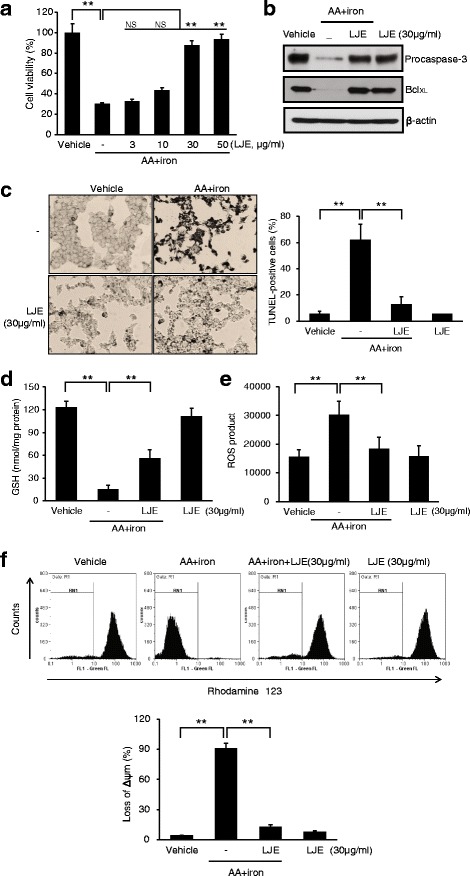
*L. japonica* extract (LJE) decreases AA + iron induced cytotoxicity in HepG2 cells. HepG2 cells were incubated with indicated dose of LJE for 1 h and later treated with 10 μM AA for 12 h, being followed by exposure to 5 μM iron for 3 h. (**a**) Cell viability was assessed by the MTT assay. (**b**) Expression of proteins associated with apoptosis was determined by western blot analysis. Equal protein loading was verified by β-actin. (**c**) The levels of apoptosis in each groups examined by TUNEL assay. Representative images show apoptosis of HepG2 cells in vehicle control, LJE treated, AA + iron treated, and AA + iron treated with LJE groups (left). Percentage of TUNEL+ cell nuclei calculated relative to total number of cell nuclei (right). (**d**) Cellular GSH content was assessed in cells by using GSH assay kit. (**e**) Cellular reactive oxygen species production was monitored by measuring intensity of dichloro fluoresce in fluorescence. (**f**) ΔΨm depolarization monitored by FACS analysis of Rh123 staining. Relative proportions of low Rh-123 intensity (RN1 fractions) are expressed as the mean ± S.D. of three separated experiments. For panel from A to E, data represent the mean ± S.D. for the four replicates. ** *p* < 0.01

An MTT assay for cell viability indicated that LJE pretreatment (3, 10, 30, and 50 μg/ml) significantly protected cells from the potential injury induced by AA + iron. Since the maximum cell viability was achieved at 30 μg/ml of LJE, the same concentration was applied in subsequent experiments (Fig. 1a). In western blot analysis, treatment of AA + iron markedly induced decreases in the protein levels of procaspase-3 and Bcl_XL_, verifying AA + iron induction of apoptosis, which was completely blocked by LJE pretreatment (Fig. 1b). Morphological examination by light microscopy and TUNEL assay (Fig. 1c) confirmed the cytoprotective effect of LJE against the synergized toxicity of AA + iron. After treatment with LJE, positive staining located in the nucleus by AA + iron were apparently decreased (Fig. 1c). To further examine the antioxidative effects of LJE, we measured the contents of GSH and ROS. The intracellular concentration of GSH was substantially decreased by AA + iron, but was recovered by LJE treatment. In contrast, treatment with LJE alone had no effects on cellular GSH levels (Fig. 1d). The ROS generation assay using DCFH-DA indicated that LJE treatment effectively abrogated increases in ROS production caused by AA + iron (Fig. 1e). The effect of LJE on AA + iron-induced loss of mitochondrial membrane potential (ΔΨm) monitored by FACS analysis of Rho123 staining (Fig. 1f). Rho123 fluorescence intensity was not significantly altered in LJE-treated cells compared to untreated controls, though AA + iron markedly reduced rhodamine fluorescence, indicating the loss of ΔΨm (Fig. 1f). LJE treatment significantly restrored the loss of ΔΨm caused by AA + iron (Fig. 1f).

### Fucoxanthin (FX) ameliorates AA + iron-induced cytotoxicity

**Fig. 2:**
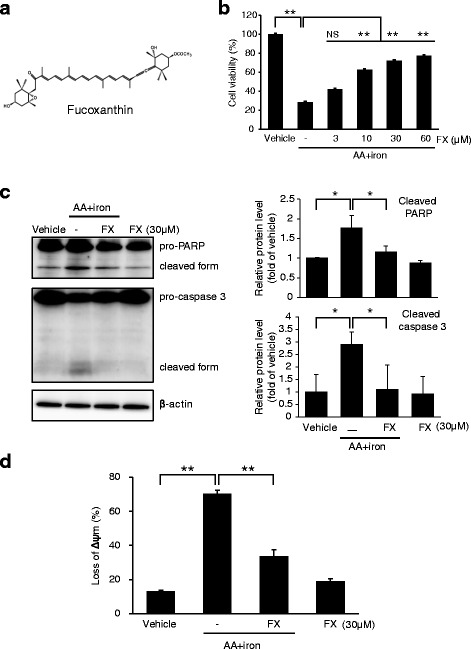
Fucoxanthin (FX) ameliorates AA + iron-induced cytotoxicity. HepG2 cells were incubated with indicated dose of FX for 1 h and later treated with 10 μM AA for 12 h, being followed by exposure to 5 μM iron for 3 h. (**a**) The chemical structure of FX. (**b**) The effects of FX on cell viability was assessed by the MTT assay. (**c**) Expression of proteins associated with apoptosis was determined by western blot analysis. Equal protein loading was verified by β-actin. (**d**) ΔΨm depolarization monitored by FACS analysis of Rh123 staining. Relative proportions of low Rh-123 intensity (RN1 fractions) are expressed as the mean ± S.D. of three separated experiments. For panel **b**, **c** and **d**, data represent the mean ± S.D. for the four replicates. ** *p* < 0.01

Next, we determined the effects of FX against oxidative stress induced by AA + iron (Fig. 2a). FX treatment inhibited death of cell induced by AA + iron, and this decrease in cell viability was recovered by pre-treatment with 30 μM of FX (Fig. 2b). In western blot analysis, cleavage of PARP and caspase-3 were strongly observed in AA + iron-treated cells, which were blocked by FX pretreatment (Fig. 2c). FX treatment significantly inhibited the change in ΔΨm caused by AA + iron (Fig. 2d). These results indicate that FX remarkably suppressed AA + iron-induced collapse of ΔΨm, consequently protecting liver cells.

### FX-induced the activation of AMPK alleviates cell damage by oxidative stress

**Fig. 3:**
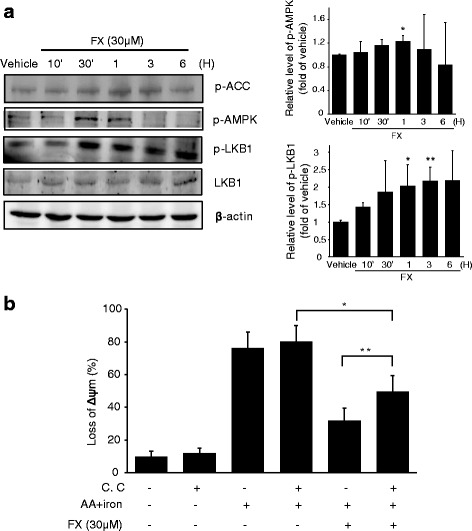
FX-induced the activation of AMPK alleviates cell damage by oxidative stress. (**a**) FX induces phosphorylation of the proteins associated with AMPK pathway, ACC, LKB1 and AMPK. Western blot analyses were performed with the lysates of cells that had been treated with 30 μM FX for the indicated time period. β-actin served as a loading control. Protein levels were presented as relative band intensities to control (vehicle treated) group. Results represent the mean ± S.D. for three separate experiments. * *p* < 0.05; ** *p* < 0.01; *** *p* < 0.001. (**b**) The effect of FX to restore ΔΨm was revered by C.C. Following treatment with 10 μM C.C for 1 h, cells were incubated with FX and/or AA + iron, and ΔΨm was evaluated with Rh123 stain by FACS. Data represent the mean ± S.D. for four replicates. * *p* < 0.05; ** *p* < 0.01

Stimulation of FX (30 μM) markedly induced the phosphorylation of AMPK (Fig. 3a). This compound also induced the phosphorylation of LKB1, an upstream kinase of AMPK, and ACC, a primary downstream target of AMPK (Fig. 3a). To determine the role of AMPK in protection of HepG2 cells by FX, we measured ΔΨm levels after treating with a chemical inhibitor of AMPK, C.C. C.C inhibited the protective effect of FX on AA + iron-induced the loss of ΔΨm in HepG2 cells (Fig. 3b). Collectively, these results suggest that FX activates the LKB1-AMPK signaling pathway, and that this activation of beneficial molecules is responsible for FX’s inhibition of mitochondria damage induced by oxidative stress.

### FX triggers AMPK-dependent cytoprotective autophagy

**Fig. 4:**
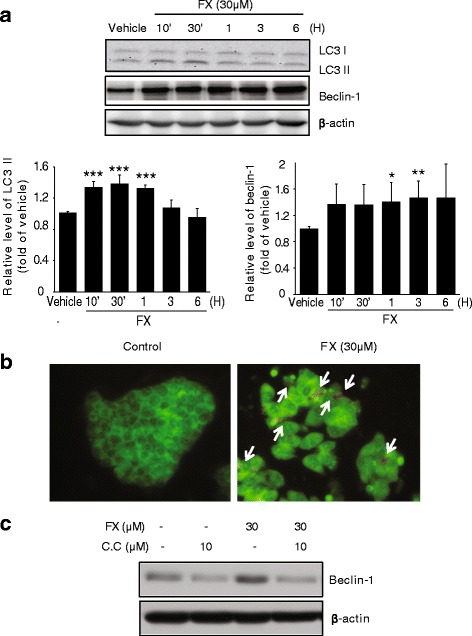
The effect of FX on autophagy induction in HepG2 cells. (**a**) FX induces time-dependent activation of the autophagy related proteins, LC3II and Becline-1. Western blot analyses were performed on the lysates of cells that had been treated with 30 μM FX for the indicated time period. β-actin served as a loading control. Protein levels were presented as relative band intensities to control (vehicle treated) group. Results represent the mean ± S.D. for four separate experiments. ** *p* < 0.01; *** *p* < 0.001. (**b**) The pictures of the fluorescence micrographs show the formation of AVOs resulting from the treatment of FX in HepG2 cells. Cells were incubated either in the presence or absence of 30 μM FX for 6 h and were stained with AO. The presence of AVOs was indicated by the red fluorescence. (**c**) Inhibition of FX-induced autophagy by C.C. Western blot analysis of Beclin-1 were performed with lysates of HepG2 cells that had been pretreated with 10 μM C.C for 1 h being followed by exposure to 30 μM FX for 1 h

**Fig. 5:**
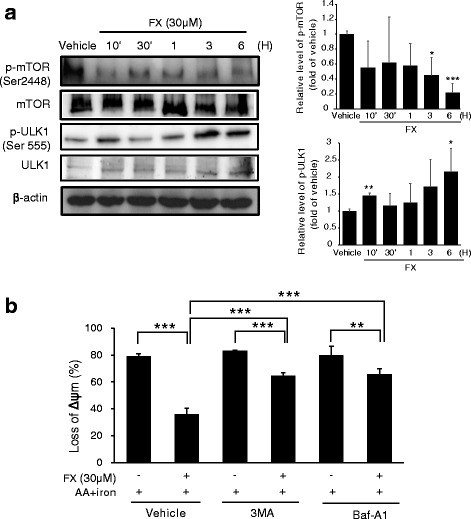
The role of AMPK activation by FX in autophagy induction. (**a**) Western blot analysis for the phosphorylated level of mTOR (Ser2448) and ULK1 (Ser555) in HepG2 cells treated with 30 μM FX for indicated time period. Results represent the mean ± S.D. for four separate experiments. * *p* < 0.05; ** *p* < 0.01; *** *p* < 0.001. (**b**) The effect of FX to restore ΔΨm was revered by 3-MA and Baf-A1. After 3-MA and Baf-A1 treatment (5 μM for 1 h, respectively), cells were incubated with FX for 1 h, being followed by the addition of AA (for 12 h) + iron (1 h). Data represent the mean ± S.D. for three replicates. ** *p* < 0.01; *** *p* < 0.001

Treatment with 30 μM FX upregulated beclin-1 and promoted the conversion of LC3 I to LC3 II as compared to the control (Fig. 4a). The AVOs were clearly observed in the HepG2 cells (red fluorescence) following treatment with FX (Fig. 4b). As shown in Fig. 4c, inhibition of AMPK activity by C.C markedly attenuated FX-induced accumulation of beclin-1, suggesting that AMPK is critical in the regulation of FX-induced autophagy. In addition, western blotting revealed that FX rapidly downregulated the phosphorylation of mTORC1 (Ser2448), which is known to negatively modulate autophagy and be inhibited by AMPK (Fig. 5a). In contrast, the phosphorylation levels of ULK1 (Ser555) was increased during FX treatment in time-dependent manner (Fig. 5a). Furthermore, we manipulated autophagy activity using 3-MA and Baf-A1 to suppress autophagy. FACS analysis of ΔΨm showed that 3-MA and Baf-A1 partially blocked the effect of FX on mitochondrial protection (Fig. 5b).

## Discussion

In our results, LJE or FX ameliorated oxidative damage induced by AA + iron on hepatocytes, as confirmed by the inhibition of cell death and the restorating the loss of ΔΨm. We also demonstrated that these hepatoprotective effects of FX can be attributed to the function of autophagy via the AMPK/mTORC1/ULK-1 axis.

To determine the capacity of LJE or FX to reduce oxidative damage, we employed an in vitro model, HepG2 cells treated with AA + iron. In liver, the level of iron is tightly regulated by the control of absorption, storage and recycling, which is critical for the protection of liver tissues as well as other organ tissues from iron-induced cellular damages [21]. However, a chronic increase of iron level in liver can result in excess ROS production and liver injury, such as steatohepatitis, fibrosis, cirrhosis, and hepatocellular carcinoma [22]. The release of AA can also be induced by an increase in oxidative stress that originates from excessive levels of iron [23]. Although prostaglandins, essential components in cellular protection, are produced from AA, excessive AA can induce extremely high levels of cellular and mitochondrial ROS, negatively influencing the functions of several processes related to mitochondrial respiration [23]. The combinatorial treatment of AA and iron can thus reduce cell viability, and this treatment may be used to test the potential of cytoprotective agents targeting mitochondria against severe oxidative stress. In this study, AA + iron successfully induced cell death, production of ROS and damage of mitochondria in HepG2 cells. However, pretreatment of LJE or FX significantly blocked the ability of AA + iron to induce similar detrimental effects in HepG2 cells.

Recent studies have shown that AMPK serves as a key regulator of hepatocytes viability under oxidative stress [10, 11, 23]. Indeed, many natural compounds, such as resveratrol, sauchinone, and isoliquiritigenin, have been reported to protect hepatocytes by inhibiting production of ROS and mitochondrial dysfunction through activation of AMPK [24–26]. In our study, FX upregulated the phosphorylation of AMPK, ACC (the downstream target) and LKB1 (the essential upstream kinase) in hepatocytes. Furthermore, the inhibition of AMPK using compound C reduced the beneficial effect of FX on mitochondria. Those results partially suggest that FX protects cells through activation of AMPK.

It is now widely accepted that AMPK induce autophagy, in turn, serving to reduce oxidative damage [12–14]. Autophagy can suppress cell death by eliminating damaged organelles or unnecessary cellular components formed from a variety of stresses, thereby playing adaptive roles to protect organisms against infections, cancer, neurodegeneration, aging, and heart diseases [15–18, 27]. The process of autophagy involves formation of double membrane vesicles (autophagosome) that enwrap portions of the cytoplasm [24]. To detect the development of AVOs, HepG2 cells treated with FX were stained with acridine orange. Our results demonstrated that the bright red fluorescence significantly increased after FX treatment, indicating the development of AVOs. Western blotting studies also demonstrated that FX induced HepG2 cell autophagy, as shown by the conversion of LC3B-I in LC3B-II and the expression of Beclin-1, indicators of autophagy [24, 28]. Furthermore, AMPK inhibition by a chemical inhibitor of AMPKα, C.C abolished the increased protein level of beclin-1 by FX. Consequently, our current findings suggest that the activation of AMPK by FX be involved in induction of autophagy in HepG2 cells.

AMPK activation promoted autophagy through direct activation of ULK1 and inhibition of mTORC1, a negative regulator of autophagy [14]. mTORC1 is important in the autophagy and its activity inhibits autophagy by ULK1 phosphorylation, which induce disassociation between ULK1 and AMPK [14]. In this study, we observed a significant increase in the levels of ULK1 and AMPK phosphorylation in response to FX. Thus, our findings indicate a model in which FX induces autophagy by activating the AMPK–ULK1–mTORC1 axis. Finally, present study showed that 3-MA and Baf-A1, the autophagy inhibitors, partially reversed the inhibitory effect of FX on AA + iron-induced mitochondrial membrane potential depolarization in HepG2 cells, suggesting the involvement of AMPK-induced autophagy as the hepatoprotective activity of FX in HepG2 cells.

**Fig. 6:**
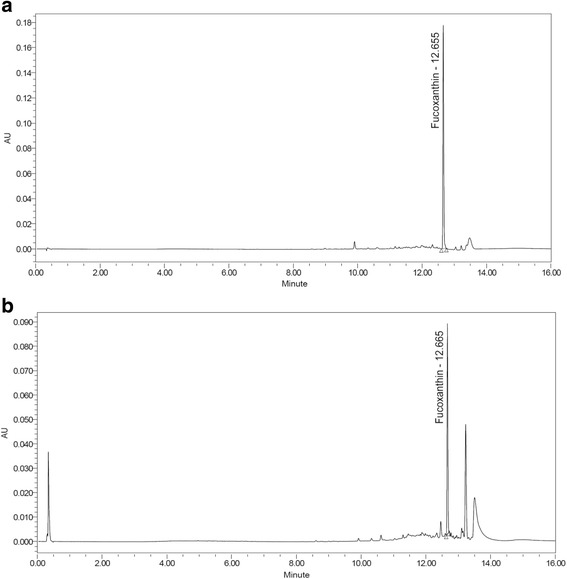
UPLC chromatogram of fucoxanthin standard (**a**) and fucoxanthin in *L. japonica* (**b**). The peak represents fucoxanthin (330 nm)

Although there are various component in *L. japonica*, in this study, we confirmed the content of FX (mass accuracy; 28.197 ppm) in *L. japonica* (Fig. 6). Recently, some report also showed that FX is known to significantly inhibit on the proliferation of HepG2 cells [29]. The result showed that both the protein degradation and transcriptional repression was responsible for cyclin D suppression by FX in HepG2 cells by inducing G1 arrest as mediated with GADD45A and MAPK pathway [29]. But, in the present study, FX showed a significantly protective effect on HepG2 cells against AA + iron-induced oxidative injury. Therefore, the active compound in the *L. japonica* and FX in the aspect of cell protection as well as their machanism remains to be further established.

## Conclusion

**Fig. 7:**
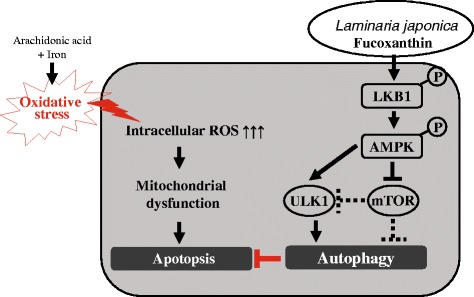
Schematic diagram showed that FX induces AMPK-mediated autophagy contributing to ameliorates oxidative stress in HepG2 cells

Our results suggested that FX could protect hepatocytes against AA + iron-induced oxidative stress and trigger autophagy, which is likely associated with the LKB1-AMPKα signaling pathway (Fig. 7). The current study also showed that FX or *Laminaria japonica* likely contributed to further understanding of its potential use as a hepatic protectant and nutraceutical.

## Abbreviations

3-MA: 3-methyladenine
AA: Arachidonic acid
ACC: Acetyl-CoA carboxylase
AMPK: AMP-activated protein kinase
ATG: Autophagy-related proteins
AVO: Acidic vesicular organelle
Bal-A1: Bafilomycin A1
Bcl-xL: B-cell lymphoma-extra large
C.C: Compound C
DCFH-DA: 2′,7′-Dichlorofluorescein diacetate
DMSO: Dimethyl sulphoxide
FBS: Fetal bovine serum
FX: Fucoxanthin
GSH: Glutathione
LC3: Microtubule-associated protein 1 light chain 3
LJE: *L. japonica* extract
LKB1: Liver kinase B1
mTOR: mammalian target of rapamycin
MTT: 3-(4,5-dimethylthiazol-2-yl)-2,5-diphenyl-tetrazolium bromide
PARP: Poly (ADP-ribose) polymerase
PBS: Phosphate-buffered saline
Rh123: Rhodamine 123
ROS: Reactive oxygen species
TUNEL: Terminal deoxynucleotidyl transferase dUTP nick-end labeling
ΔΨm: mitochondrial membrane potential
